# VBNC, previously unrecognized in the life cycle of *Porphyromonas gingivalis?*

**DOI:** 10.1080/20002297.2021.1952838

**Published:** 2022-01-13

**Authors:** A Progulske-Fox, SS Chukkapalli, H Getachew, WA Dunn, JD Oliver

**Affiliations:** a Department of Oral Biology, Center for Molecular Microbiology, University of Florida, Gainesville, FL, USA; b Center for Molecular Microbiology, University of Florida, Gainesville, FL, USA; c Department of Anatomy and Cell Biology, University of Florida, Gainesville, FL, USA; d Department of Biological Sciences, University of North Carolina, Charlotte, NC, USA

**Keywords:** VBNC, *P. gingivalis*, chronic infections, oral bacteria, resuscitation

## Abstract

Bacteria are exposed to stresses during their growth and multiplication in their ecological systems to which they respond in multiple ways as expert survivalists. One such response mechanism is to convert to a viable but not culturable (VBNC) state. As the name indicates, bacteria in the VBNC state have lost their ability to grow on routine growth medium. A large number of bacteria including many pathogenic species have been reported to be able to enter a VBNC state. VBNC differs from culturable cells in various physiological properties which may result in changes in chemical resistance, adhesion, cellular morphology, metabolism, gene expression, membrane and cell wall composition and/or virulence. The ability of VBNC bacteria to return to the culturable state or resuscitate, when the stressor is removed poses a considerable threat to public health. There have been few publications that overtly describe the ability of oral pathogenic species to enter the VBNC state. However, the presence of VBNCs among oral pathogens such as *Porphyromonas gingivalis* in human chronic infections may be an important virulence factor and have severe implications for therapy. In this review, we intend to i) define and summarize the significance of the VBNC state in general and ii) discuss the VBNC state of oral bacteria with regard to *P. gingivalis*. Future studies focused on this phenomenon of intraoral VBNC would provide novel molecular insights on the virulence and persistence of oral pathogens during chronic infections and identify potential novel therapies.

## Introduction

Bacterial species have evolved multiple mechanisms to survive ecological, nutritional and chemical stresses. Among these is to enter a viable but not culturable (VBNC) state [1]. As the name indicates, bacteria in the VBNC state have lost the ability to grow on routine bacteriologic media [2]. Despite their inability to grow on usually permissive media, VBNC cells are, in fact, not dead nor spores, although the differences between VBNC and culturable cells are considerable. These include differences in chemical resistance, adhesion properties, cellular morphology, metabolism, gene expression, membrane and cell wall composition and virulence properties [2]. Yet, VBNC cells retain complete membranes, maintain the ability to synthesize mRNA, are metabolically active, carry out respiration, and take up and incorporate amino acids into proteins [2]. Researchers globally have identified about 100 species of bacteria that can enter the VBNC state. The VBNC state is also observed to be present in yeasts such as *Saccharomyces cerevisiae* (Salma M et al., PLOS one 2013), wine spoilage yeast *Brettanomyces* (Serpaggi V et al., 2012 Food Microbiolo) as well as in fungi (*Cryptococcus neoformans* Hommel B et al., PLOS pathogens 2019). At least one bacterial species has been reported to express a virulence factor during the VBNC state [3].

The stressful conditions that induce bacterial cells to enter the VBNC state are known for numerous species and have been found to vary not only among species but also strains. Some of the more common stressors described to date include starvation, antibiotic treatment and temperature changes [2]. Most importantly, VBNC bacteria have the ability to return to the culturable state or resuscitate, when the stressor is removed or negated. The length of time during which bacteria in the VBNC state can be resuscitated, the resuscitation window, varies widely among species. For example, it has been reported to be as short as 3 days for *Vibrio vulnificus* [4] or as long as 11 years for *Citrobacter freundii* [5].

Pathogenic VBNC bacteria initially gained attention due to their ability to exist at non-detectable thresholds by traditional water and food safety techniques. Several pathogens in this state may contribute initially to non-apparent infections, which are later followed by a manifestation of the disease. For example, *Mycobacterium tuberculosis* has been demonstrated to exist in a VBNC state and latent TB reactivation may be a attributed to resuscitation from the VBNC state [6]. Importantly, as reported by Oliver [7], the VBNC state of bacteria including *Enterococcus faecalis, Escherichia coli, Haemophilus influenza, Helicobacter pylori* and *Mycobacterium smegmatis* was significantly associated with increased resistance to antibiotics.

## VBNC: a distinct physiological state of survival

The VBNC state was first discovered in 1982 in *E. coli* and *Vibrio cholerae* cell [1] and to date has been reported to exist in nearly 100 diverse bacterial species living in a wide range of environments [2,8,9]. The VBNC state represents a distinct physiological state. These bacteria can be identified by various detection methods to be alive and capable of subsequent resuscitation back to a culturable state. In general, the majority of bacterial species that have been observed to exist in the VBNC state are free living and exist in broad environmental conditions including food, water, and soil.

The VBNC state of bacteria was traditionally referred to as a dormant bacterial state. However, it is now clear that there exists a difference between VBNC and dormant stages based on their metabolic activity. The VBNC state is characterized by a metabolic activity that is always measurable, whereas during the dormant state, this activity declines to levels that are lower than the level of detection (Table 1). Thus the VBNC state is now widely accepted as a distinct physiological state of survival of bacteria [2,10]. This physiological state is not only a mechanism employed by bacteria to support their long-term survival during unfavorable conditions but may also contribute to cellular decay as a mechanism of preserving specific features of viable cells which results in an ‘intentional’ net loss of culturable characteristics.

**Table 1.** Characteristic differences between VBNC, persistent and culturable states

VBNC	Persistent	Culturable
Not culturable on normal culture medium	Still culturable	Can grow on normal culture medium
Lowered metabolism	Active metabolism	Highest metabolism
High antibiotic tolerance	Antibiotic tolerance	Antibiotic sensitive
Highly tolerant to stress conditions	Can tolerate stress moderately	Do not tolerate stress conditions
Takes a longer time to grow after stress conditions are removed	Quickly regain growth after stress conditions are removed	Lose growth under stress conditions well
Changes in cellular morphology	No changes in cellular morphology	No change in cellular morphology

## Changes in cellular morphology during the VBNC state

Cellular morphology is a critical aspect of the phenotype of a cell. Since the cell wall/peptidoglycan determines the shape of the cell, as it enables the tensile strength and diffusion barriers that are necessary to attain a particular shape, any differences in the cell wall composition may affect the cellular morphology. This occurs in some VBNC bacterial cells as many have shown differences in cellular morphology such as cell dwarfing and rounding [11,12]. It is likely that a reduction in cell size is a strategy to limit the energy demands of VBNCs [13–15]. For example, *Campylobacter* spp. change from the characteristic spiral shape during normal exponential phase growth to a coccoid shape during transition to the VBNC state [16]. Similarly, *Burkholderia pseudomallei* and *V. cholerae* cells were also observed to change morphology from their characteristic rod to cocci shape during transition to the VBNC [17,18]. However, these morphological changes commonly seen in VBNC cells are not exclusive to VBNC cells, as similar changes are observed in non-VBNC cells that live under stressful conditions. Hence, a morphological change alone cannot be used to define the VBNC state [19].

## Metabolism and gene expression in VBNC cells

### Induction of the VBNC state

Bacterial cells undergo a change in their physiological state to VBNCs in response to various conditions including but not limited to environmentally unfavorable conditions [7], starvation of nutrients [12], temperature fluctuations [20], incubation outside optimal pH [21], changes in osmotic concentrations [22], differences in oxygen concentrations [23], heavy metal exposure [24,25], and importantly on exposure to antibiotics. Since there is a broad range of bacterial species that can enter the VBNC state, it is likely that diverse regulatory mechanisms control this state among these species. Some genes that are reportedly involved in regulation of the VBNC state in *E. coli* and *Salmonella typhimurium* include rpoS and *oxyR*. rpoS is a sigma factor that is essential for survival in stationary phase as well as during stress responses [26,27]. *oxyR* is a l*ysR*-type transcriptional regulator which has a characteristic N-terminal DNA-binding domain that is known to regulate oxidative stress-related genes and plays a crucial role in the induction of the VBNC state [28,29].

The lower metabolic rate of VBNCs results in changes in proteins, fatty acids and peptidoglycan in their cell walls and membranes. For example, there was an observed shift in the outer membrane subproteome in *E.coli* VBNCs including the levels of outer membrane proteins (Omp), the 43 β-subunit antigen, TolC, and OmpT while there was a shift in 106 proteins in VBNCs resulting from exposure to natural seawater and light [30]. Significantly, Omp W was found to be highly induced in VBNC cells [22]. During transition of *V. vulnificus* cells to the VBNC state, the levels and composition of unsaturated fatty acids increased with a major shift towards fatty acids with less than 16 carbons and increases in hexadecanoic, hexadecenoic, and octadecanoic acids [31]. Further, an increase in peptidoglycan cross-linking was also observed in *E. faecalis* VBNCs compared to culturable cells [32].

VBNC cells also have a variable gene expression profile compared to their culturable counterparts. For example, 58 genes related to regulatory functions, cellular processes, energy metabolism as well as transport and binding were all induced by more than 5-fold in *V. cholerae* [33], while another study found a reduction in 16S rRNA as well as the mRNA levels of *tuf, rpoS, and relA* genes that are responsible for protein synthesis and stress responses [34].

These regulatory and physiological changes produce VBNCs that exhibit strong resistance to various physical, chemical and temperature stress conditions. For example, VBNC cells of *V. vulnificus* were observed to be resistant to mechanical destruction by sonication [35], while those of *M. smegmatis* were found to be more resistant to high temperatures [36]. VBNC cells of *Vibrio parahaemolyticus* [37], *Campylobacter jejuni* [38] and *E. faecalis* [39], were also found to show greater levels of resistance against low salinity, ethanol, and chlorine, respectively.

### Detection of VBNC

An array of viability markers have been used to differentiate viable and VBNC cells. The most common method is differential staining combined with direct microscopic enumeration, the LIVE/DEAD® Bac Light™ assay. This assay employs two fluorescent dyes, Syto 9 and propidium iodide, which have variable cell permeability characteristics that can differentiate cells with different membrane integrities [21].

The p-iodonitrotetrazolium violet (INT) assay, is another method and is based on the activity of an electron transport system [40]. As only viable cells can undergo metabolic activity and respiration, this assay can be used to differentiate the VBNC and dead cells [15]. INT is a soluble tetrazolium salt which competes with oxygen for electron acceptance and on reduction turns to an insoluble formazan complex in metabolically active cells. Hence, the formation and accumulation of formazan in cells, which is observed as a dark red precipitate under a microscope, indicates viable cells. The tetrazolium salt, 5-cyano-2,3-ditolyltetrazolium chloride (CTC) or the BacLight™ RedoxSensor™ Green have also been used [20,41].

Untargeted metabolomics is being developed as a new method to identify the VBNC state [42,43]. With the increased metabolomics research capabilities, metabolomics technology is continuously evolving and has the potential to identify precise metabolic markers that could easily distinguish between culturable and VBNC cells based on the presence of metabolomic markers in the future [44]. Diphenyleneiodonium (DPI) treatment was used as an inducing agent to cause rapid transition in mycobacteria from an active state into a viable, but non-cultivable state, and comparing their characteristics with dormant phenotypes using untargeted metabolomics [43].

### Resuscitation of VBNC cells to a culturable state

The term ‘resuscitation’ was first used to describe the recovery of non-culturable *Salmonella enteritidis* cells with the subsequent addition of HI broth. Two decades later, this phenomenon was again reported by Baffone et al. [45], who defined it as the state of reversal of metabolic and physiological changes that characterize VBNC cells. This phenomenon is hugely significant in the bacterial life cycle and poses a significant threat to human health. This was first realized for diseases caused by pathogens in the food or water supply since the safety of water and foods is routinely determined by plate counts of culturable cells. Consequently the presence of VBNC cells in a water or food sample results in spuriously low bacterial (or zero) plate counts which then is erroneously interpreted that the water/food is safe for consumption.

Another common threat, especially in the case of several host-associated human pathogens, occurs when an antibiotic is prescribed to treat an infection. The presence of an antibiotic can induce a bacterium to enter the VBNC state. In some cases, when the antibiotic is discontinued, the VBNC bacteria resuscitate, allowing the infection to reoccur. There is recent evidence that suggests some intracellular pathogens can enter the VBNC state inside their host cells, thus ‘hiding’ from the cell’s antibacterial mechanisms and allowing them to persist. This ability of a bacterial species to enter a state of VBNC has been linked to the survival and pathogenicity of multiple host-associated species including *Pseudomonas aeruginosa* and *M. tuberculosis* [2,46]. For example, it is now recognized that the chronicity of tuberculosis is due to *M. tuberculosis* existing in a VBNC state.

The signaling mechanisms of resuscitation are not well understood because there are no readily available methods/techniques that can distinguish between culturable cells and those that arise from resuscitation. Thus far documented resuscitation has been reported only in approximately half of the human pathogens that are known to exist in the VBNC state. Thus mechanisms that govern resuscitation of pathogenic VBNC cells are mostly unknown which complicates more fully understanding of host-bacterial interactions and outcomes.

The issue becomes further complicated because of the ‘resuscitation window’, as first proposed by Pinto et al. [19] and defined as the time period during which VBNC cells retain their ability to resuscitate under normal suitable stimuli. It was observed that on exposure to inducing conditions, different cells in a population enter into a VBNC state at different times. Based on this, a hypothesis was proposed which stated that VBNC cells belonging to the same species will have a fixed resuscitation window, in that the older VBNC cells will lose their resuscitation ability earlier than the younger VBNC cells, resulting in a reduction of total resuscitable cells over time. Data from multiple studies have supported this hypothesis, as a reduction in the number of resuscitable cells over time was found in both *V. cholerae* and *E. faecalis* [18,47].

Various factors have been tested as a stimulus for resuscitation including a shift in temperature [48], the use of gas mixtures [49], the addition of amino acids [9], rich media [50], supernatant from spent culture medium [51–53], the addition of fresh host cells [54], and the addition of antioxidants to culture plates, among others [11,47,55]. It was also shown that the addition of specific compounds such as amino acids, resuscitation-promoting factors (Rpfs) and autoinducers [9,51,53] could resuscitate some species of VBNC cells.

## The differences between persistence and VBNC states

The VBNC and persistence states were independently described decades apart but the phenotypic similarities between them require definitions that distinguish VBNC cells from persistent cells. Persistence occurs when the majority of a population of the cells are susceptible to an antibiotic, while a subpopulation of the cells are tolerant to the antibiotic. Thus persistent cells can be considered as phenotypic variants in a population [56]. Similar to persistent cells, VBNC cells also show antibiotic tolerance, and tolerance to various other stress conditions including exposure to heavy metals, high and low temperatures, fluctuation in pH, oxidative and osmotic challenges, and ethanol [57]. Hence, the line of distinction between ‘persistence’ and ‘viable but non culturable’ cells is quite thin. However, a crucial difference is their resuscitation dynamics. While persisting bacteria are characterized by a typically shorter resuscitation period and ability to grow on nutrient media immediately following the removal of antibiotics, VBNC cells require a longer resuscitation period after removal of the inducing stress. In addition, VBNC cells require an external stimulus in order to restore metabolic competence and to repair their damaged proteins that are necessary for their growth.

Bamford and colleagues, using a single-cell imaging technique, proposed a new identification and isolation system for persisters and VBNC cells which is based on their differential promoter activity expression [58]. This method has the potential to reveal a deeper understanding of the molecular changes that cells undergo during their transition from active growth to VBNC.

## VBNC in the oral cavity?

The oral cavity can be defined as a mixture of distinct dynamic ecological habitats that support growth of a specific microbial community because of their characteristic biological features. To date, there are no publications that overtly describe the ability of *P. gingivalis* or any other oral species to enter the VBNC state, with the exception of *E. fecalis* for which the oral cavity is a secondary habitat. However, we propose such a state not only occurs but is key to the survival of *P. gingivalis in vivo*, thereby contributing to chronic infections.

## *P. gingivalis* and VBNC

There are few publications that provide support for this hypothesis. First, laboratories worldwide attempted for many years to culture periodontal bacteria from diseased atherosclerotic vessels without success. This was partially in response to the criticism that studies that identified genomic DNA of oral pathogens in these diseased tissues did not prove the presence of viable pathogens, only the presence of the DNA, which could have been transported to the diseased site by macrophages. Then, in 2005, our laboratory published the first proof of the presence of viable *P. gingivalis* in diseased tissues [59]. This was accomplished by adding carotid atherosclerotic plaque homogenate directly to human cardiovascular aortic endothelial cells (HCAEC) and visualizing the presence of *P. gingivalis* within the ECV-304 cells using species-specific fluorescently tagged antibodies combined with deconvolution microscopy. This determination was crucial, as dead *P. gingivalis* cannot invade these nonphagocytic cells. Thus, blood agar did not contain the signal for growth and division but host cells did. The intriguing question then became, what was it about *P. gingivalis in vivo* that was different from *P. gingivalis* cultured *in vitro*?

A second important publication, by Li et al. [60], demonstrated that intracellular *P. gingivalis* strain W83 in either endothelial or smooth muscle cells could be enumerated by colony counting, for up to 48 hours of co-culture. However after 48 hours, the numbers of colonies detected on plates decreased to essentially zero. This occurred at the same time numerous intact *P. gingivalis* were visualized inside the cells by microscopy. Furthermore, when the infected cells were lysed and added to uninfected cells, there was a significant number of colonies culturable on blood agar plates. These results suggested that the intracellular *P. gingivalis*, although not culturable after 48 hours, were nevertheless viable and the addition of fresh uninfected host cells provided a signal for transformation into the culturable state.

Finally, Kozarov and coworkers were subsequently able to culture and isolate additional strains of *P. gingivalis* from atheroma plaques using the co-culture approach (Kozarov and personal communication). We have spent the ensuing years contemplating the state of *P. gingivalis* in human atheromatous tissues that prevents them from growing on normally permissive medium. Our hypothesis is that they become VBNCs while residing intracellularly *in vivo.*

*P. gingivalis* has been linked to a large number of other systemic chronic diseases, some of which include cardiovascular diseases, rheumatoid arthritis, preterm birth, Alzheimer’s disease, and several types of cancers. In a recent observation by Ursula H et al., 2020, *P. gingivalis* was observed to exist in a VBNC state in neuronal cultures *in vitro*. We propose that the chronicity of *P. gingivalis* associated infections, both oral and systemic, is due to the ability of *P. gingivalis* to enter the VBNC state and subsequently periodically resuscitate within cells of the oral cavity or elsewhere within the body.

Most investigations of the VBNC state to date have studied bacteria in freeliving conditions, without the complication of the bacterial cells being intracellular. However, it was recently reported that intracellular *Listeria monocytogenes* changes from the cytoplasmic motile lifestyle, in which it uses host cell actin to transit from one host cell to another, to a state in which the bacteria lose their ability to interact with actin and instead localize within LAMP1 positive lysosomal-like vacuoles [61]. Surprisingly, the bacteria localized to lysosomes are able to resist degradation and enter into a nonreplicative state, determined to be VBNC. The authors also reported that *L. monocytogenes* can ‘revert’ to its normal state of replication upon co-culturing with the infected host cells. The authors suggest that VBNC represents a state of persistence that can then result in the asymptomatic carriage of this bacterium by human hosts, thereby lengthening the incubation period of listeriosis and promoting *L. monocytogenes* survival/resistance.

## Evidence of the VBNC state and *P. gingivalis*

**Figure 1.**
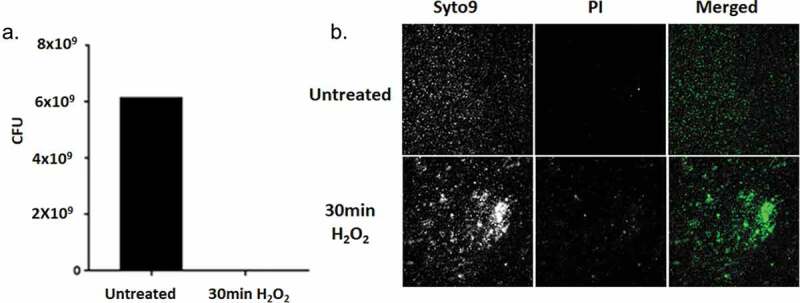
(a) Viability of PgW83 after 30 min exposure to oxidative stress (H2O2) in comparison to the untreated control. Following the 30 min exposure, the cultures were serially diluted and plated on blood agar plates. (b) PgW83 cultures demonstrating cells in the VBNC state as detected with Live/Dead BacLight staining, after exposure to oxidative stress. Following treatment, PgW83 was no longer culturable. However, viable PgW83 were visible, according to Syto9 staining. Thus PgW83 transitioned to the VBNC state in response to oxidative stress.

Based on the studies cited above, we began an investigation of the possibility of P. *gingivalis* entering into a VBNC state. Our first experiments were limited to *in vitro* conditions. We reasoned that oxidative stress would be one condition to investigate, since *P. gingivalis* encounters oxidative stress *in vivo*. To this end, oxidative stress was induced by the addition of 10 mM hydrogen peroxide to stationary P. *gingivalis* cultures. After 30 min of exposure, the cultures were plated to determine colony-forming units (CFUs) and viability was assayed using the LIVE/DEAD BacLight Bacterial Viability Kit. As can be seen in Figure 1(a), no CFUs were detected on blood agar plates. However, LIVE/DEAD backlight staining demonstrated the presence of numerous viable bacteria (Figure 1(b)), indicating that *P. gingivalis* strain W83 had indeed entered the VBNC state after exposure to oxidative stress.

**Figure 2.**
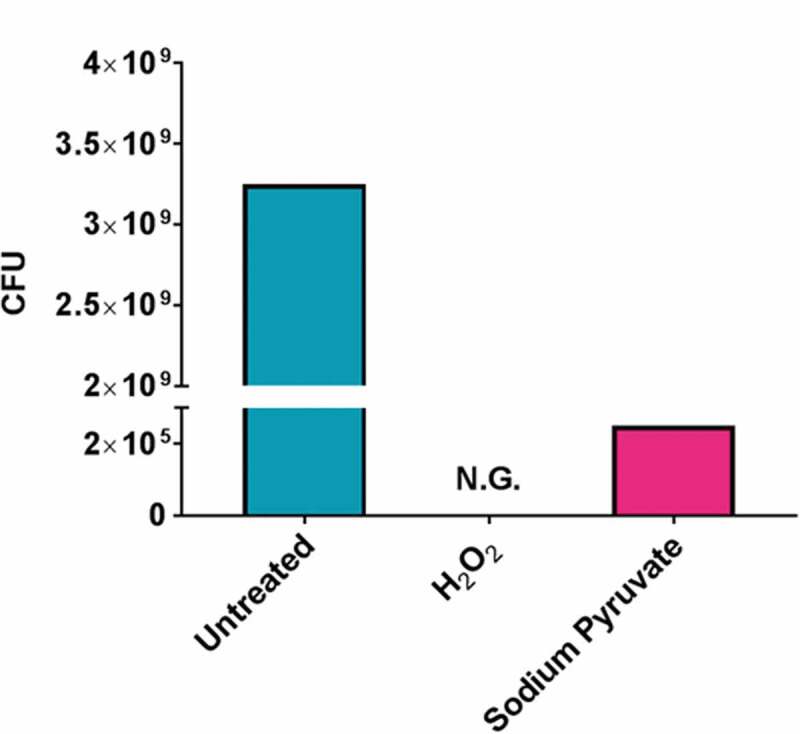
Resuscitation of PgW83 in the VBNC state to the culturable state. Following H2O2 treatment, PgW83 was no longer culturable but viable according to Syto9 staining indicating PgW83 was in the VBNC state (see Figure 1). When treated with sodium pyruvate following H2O2 stress, PgW83 was viable (Syto9 staining not shown) and culturable on blood agar plates indicating the cells have been resuscitated.

We next attempted resuscitation of the VBNC cells that were exposed to H_2_O_2_ by the addition of 0.6 mM sodium pyruvate for 60 min. The rationale behind the selection of sodium pyruvate was due to the cytoprotection ability of it against oxidative stress. These cultures were then plated and viability was determined. As can be seen in Figure 2, we observed that on exposure to sodium pyruvate the VBNC bacteria became culturable and viable, indicating that W83 was resuscitated from the VBNC state by the addition of sodium pyruvate.

**Figure 3.**
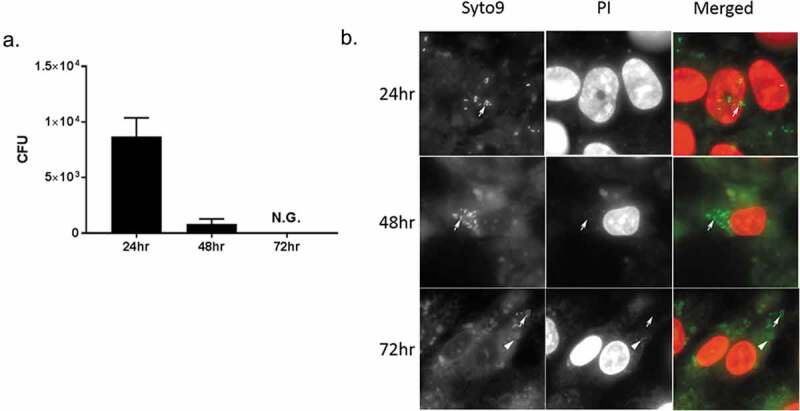
(a) Entry of PgW83 into a VBNC state in HCAEC after 72 hours post infection. HCAECs were exposed to PgW83 at 100 moi. To ensure that only intracellular bacteria were enumerated, HCAECs were treated with antibiotics at 1.5 hours post infection. After 24, 48, and 72 hours, the HCAECs were lysed and PgW83 CFUs quantified by culture on blood agar plates. While PgW83 was culturable at 24 and 48 hours, by 72 hours, this was no longer the case. (b) Micrographs of PgW83 infected HCAECs stained with Live/Dead stain at 24, 48, and 72 hours post infection. Arrows indicate live bacteria and arrowheads indicate dead bacteria. Viable (arrows) and culturable PgW83 were detected at 24 and 48 hours post infection. At 72 hours, viable PgW83 (arrows) were observed, but not culturable consistent with the VBNC state.

Subsequently, we investigated the possibility that intracellular *P. gingivalis* strain W83 could convert to the VBNC state. Using our human Coronary Artery Endothelial Cell (HCAEC) model, we observed that after 72 hours of infection, no viable *P. gingivalis* could be detected by plate counting (Figure 3(a)). However, again using live/dead staining, we determined that the intracellular bacteria were indeed alive (Figure 3(b)). Together, these data suggested that *P. gingivalis* strain W83 can exist in a VBNC state not only under *in vitro* conditions but also in intracellular conditions. Also, when the VBNC inducing condition is removed, *P. gingivalis* W83 can resuscitate.

Furthermore, we have identified a number of putative PgW83 homologues of gene products that have been used as VBNC markers or required for the VBNC or resuscitation states in other bacteria (Table 2). PorR, Pg1393, and Pg0575 show homology to the VBNC markers in *E. coli* and *E. faecalis*. RpoD in PgW83 is a putative homologue of rpoS, an RNA polymerase sigma factor, required for *E. coli* to enter the VBNC state. PgW83 has a comparable two-component signaling system (Pg1797 and Pg1089), which has been proposed to be a sensor to initiate the VBNC state in *E. coli*. PgW83 also has homologues to two *Vibrio* genes required for the VBNC state. Finally, we have identified possible PgW83 homologues to resuscitation genes characterized in *Rhodococcus, Mycobacterium*, and *Vibrio*.

**Table 2.** Bacterial genes used as VBNC markers or required for the VBNC or resuscitation states

Gene	Species/genus	Stressor*	Putative PgW83 Homologues**	Putative Function	References
VBNC State					
*rpoS*	*E. coli, Salmonella*	Starve,Osmol	RpoD (Pg0594)	RNA polymerase, sigma factor	[2,26,62]
*oxyR*	*Vibrio*	Oxidative,Temp	OxyR (Pg0270)	Redox-sensitive transcription factor	[2,63]
*ahpC2*	*Vibrio*	Temp	Pg0618	Alkyl hydroperoxide reductase C	[64]
*perR*	*Campylobacter*	Oxidative	Fur (Pg0465)	Ferric uptake transcription factor	[65]
*envZ*	*E. coli*	pH,Starve,	Pg1797	Sensor histidine kinase	[2,66]
		Osmol			
*ompR*	*E. coli*	pH,Starve,	PprY (Pg1089)	DNA response regulator	[2,66]
		Osmol			
VBNC Resuscitation					
*rpfA-E*	*Mycobacterium*	Starve, Anoxia	none	Resuscitation-promoting factors	[2,19,23,67,68]
*rpf*	*Rhodococcus*	Starve, Temp	Pg0149	Resuscitation-promoting factor	[69,70]
*pknB*	*Mycobacterium*	Anoxia	Pg0778	Ser/Thr protein kinase	[2,71]
*yeaZ (tsaB)*	*Vibrio*	Temp	Pg1724	tRNA threonylcarbamoyladenosine synthesis	[68,72]
VBNC Markers					
*rfbE*	*E. coli*	Starve	porR (Pg1138)	GDP-perosamine synthase	[73]
*stx1,2*	*E. coli*	Starve	none	Shiga toxin	[73,74]
*pbp5*	*Enterococcus*	Temp, Anoxia	Pg1393/Pg0575	D-alanyl-D-alanine decarboxypeptidase	[47]

* Starve: deionized water or nutrient-free salt solution Osmolarity: 7% NaCl

Oxidative: 1–2 mM H_2_O_2_ or 100 µM cumene hydroperoxide or 100 µM menadione; Anoxia: oxygen depletion (nonshaking or sealed flask)

Temperature: 4–5°C pH: 8.3

**The PgW83 putative homologues were identified using tblastn methodology at the NCBI site (https://blast.ncbi.nlm.nih.gov/Blast.cgi)

These findings offer new insights into the lifecycle of *P. gingivalis* strain W83 and likely other strains of *P. gingivalis* as well. We now are aware that *P. gingivalis* strain W83 appears to have the necessary genes for the VBNC state and resuscitation and is capable of entering a VBNC state and can resuscitate, once an appropriate environment/signal is present, to a culturable state. These observations are significant not only in terms of the pathobiology of *P. gingivalis* strain W83 but also call into question the conclusions of previously published studies of trafficking of *P. gingivalis* within host cells. The previously published investigations should be reevaluated and reinterpreted, since the vast majority of such studies used only CFU enumeration to determine live bacteria within the host cells.

## Other oral bacteria

It is almost certain that other oral bacterial species can enter and resuscitate from a VBNC state. *Streptococcus mutans, Streptococcus pneumoniae, Streptococcus pyogenes*, and *Streptococcus sanguinus* were also reported to display phenotypes that closely resemble the description of VBNC states. *E. fecalis* for which the oral cavity is a secondary habitat is observed to adopt the VBNC state in response to environmental conditions. As additional studies are done, we expect that a variety of oral bacteria will also demonstrate that the VBNC state is part of their lifestyle. Thus investigation of the VBNC state in oral bacterial pathogens is vital to more completely define and characterize all phases of their lifestyle, thereby more fully understanding their virulence/survival mechanisms. This understanding of a previously unknown lifestyle should result in new and novel preventative approaches and treatments for oral and other diseases. For example, the identification of the molecular determinants required for the transition of *P. gingivalis* to the VBNC state and/or resuscitation during infection could lead to the development of therapeutics that target the chronicity of infections by this bacterium. In addition to antimicrobials, disinfectants also have been shown to trigger entry into the VBNC state. It is thus entirely possible that disinfecting agents used in oral care, e.g. in endodontics may induce the associated oral bacteria to enter the VBNC state. The chronic presence of VBNC of oral bacteria may have severe implications for therapy for infections of the oral cavity as well as infections caused by these pathogens at various and multiple sites within the body.

## Future perspectives

The precise role of the VBNC state in bacterial pathogenesis is yet to be elucidated. It is possible that its role and significance differ from bacterium to bacterium. It will be crucial to understand the role of VBNCs in evasion of host immune detection. By this publication, we seek to convey to the community studying the oral microbial populations that there is a need to initiate studies to further investigate the significance, role and molecular signaling mechanisms of the VBNC and resuscitation states not only in *P. gingivalis* but other pathogens as well. More importantly, future studies of this previously unknown and unappreciated stage of the life cycle of oral pathogens will define mechanisms that can be targeted for novel antimicrobial agents designed to interfere with the VBNC and/or resuscitation states. The identification of such drug targets would have the potential to prevent the persistence and chronicity of not only *P. gingivalis* infections, but those of other bacterial species involved in chronic diseases.

## References

[1] Xu HS, Roberts N, Singleton FL, et al. Survival and viability of nonculturable *Escherichia coli* and *Vibrio cholerae* in the estuarine and marine environment. Microb Ecol. 1982;8(4):313–10. 24226049 10.1007/BF02010671

[2] Li L, Mendis N, Trigui H, et al. The importance of the viable but non-culturable state in human bacterial pathogens. Front Microbiol. 2014;5:258. 24917854 10.3389/fmicb.2014.00258 PMC4040921

[3] Alleron L, Khemiri A, Koubar M, et al. Vbnc *Legionella pneumophila* cells are still able to produce virulence proteins. Water Res. 2013;47(17):6606–6617. 24064547 10.1016/j.watres.2013.08.032

[4] Oliver JD, Bockian R. *In vivo* resuscitation, and viru- lence towards mice, of viable but nonculturable cells of *Vibrio vulnificus*. Appl Environ Microbiol. 1995;61(7):2620–2623. 7618873 10.1128/aem.61.7.2620-2623.1995 PMC167533

[5] Dhiaf A, Bakhrouf A, Witzel KP. Resuscitation of eleven-year vbnc citrobacter. J Water Health. 2008;6(4):565–568. 18401122 10.2166/wh.2008.131

[6] Mukamolova GV, Turapov O, Malkin J, et al. Resuscitation-promoting factors reveal an occult population of tubercle bacilli in sputum. Am J Respir Crit Care Med. 2010;181(2):174–180. 19875686 10.1164/rccm.200905-0661OC PMC2809243

[7] Oliver JD. Recent findings on the viable but noncul- turable state in pathogenic bacteria. FEMS Microbiol Rev. 2010;34(4):415–425. 20059548 10.1111/j.1574-6976.2009.00200.x

[8] Highmore CJ, Warner JC, Rothwell SD, et al. Viable- but-nonculturable *Listeria monocytogenes* and *Salmonella enterica* serovar *thompson* induced by chlorine stress remain infectious. MBio. 2018;9(2):2. 10.1128/mBio.00540-18 PMC5904417 29666286

[9] Pinto D, Almeida V, Almeida Santos M, et al. Resuscitation of Escherichia coli vbnc cells depends on a variety of environmental or chemical stimuli. J Appl Microbiol. 2011;110(6):1601–1611. 21447017 10.1111/j.1365-2672.2011.05016.x

[10] Oliver JD. The viable but nonculturable state in bacteria. J Microbiol. 2005;43:93–100. 15765062

[11] Adams BL, Bates TC, Oliver JD. Survival of *Helicobacter pylori* in a natural freshwater environment. Appl Environ Microbiol. 2003;69(12):7462–7466. 14660399 10.1128/AEM.69.12.7462-7466.2003 PMC310012

[12] Cook KL, Bolster CH. Survival of *Campylobacter jejuni* and *Escherichia coli* in groundwater during pro- longed starvation at low temperatures. J Appl Microbiol. 2007;103(3):573–583. 17714390 10.1111/j.1365-2672.2006.03285.x

[13] Biosca EG, Amaro C, Marco-Noales E, et al. Effect of low temperature on starvation-survival of the eel pathogen *Vibrio vulnificus* biotype 2. Appl Environ Microbiol. 1996;62(2):450–455. 8593047 10.1128/aem.62.2.450-455.1996 PMC167812

[14] Du M, Chen J, Zhang X, et al. Retention of virulence in a viable but nonculturable *Edwardsiella tarda* isolate. Appl Environ Microbiol. 2007;73(4):1349–1354. 17189433 10.1128/AEM.02243-06 PMC1828651

[15] Rahman I, Shahamat M, Chowdhury MA, et al. Potential virulence of viable but nonculturable *Shigella dysenteriae* type 1. Appl Environ Microbiol. 1996;62(1):115–120. 8572688 10.1128/aem.62.1.115-120.1996 PMC167780

[16] Thomas CH, Collier JH, Sfeir CS, et al. Engineering gene expression and protein synthesis by modulation of nuclear shape. Proc Natl Acad Sci U S A. 2002;99(4):1972–1977. 11842191 10.1073/pnas.032668799 PMC122304

[17] Inglis TJ, Sagripanti JL. Environmental factors that affect the survival and persistence of *Burkholderia pseudomallei*. Appl Environ Microbiol. 2006;72(11):6865–6875. 16980433 10.1128/AEM.01036-06 PMC1636198

[18] Senoh M, Ghosh-Banerjee J, Ramamurthy T, et al. Conversion of viable but nonculturable *Vibrio cho- lerae* to the culturable state by co-culture with eukar- yotic cells. Microbiol Immunol. 2010;54(9):502–507. 20840148 10.1111/j.1348-0421.2010.00245.x

[19] Pinto D, Santos MA, Chambel L. Thirty years of viable but nonculturable state research: unsolved molecular mechanisms. Crit Rev Microbiol. 2015;41(1):61–76. 23848175 10.3109/1040841X.2013.794127

[20] Besnard V, Federighi M, Declerq E, et al. Environmental and physico-chemical factors induce vbnc state in *Listeria monocytogenes*. Vet Res. 2002;33(4):359–370. 12199363 10.1051/vetres:2002022

[21] Obc CE, Oliver JD. Effect of weak acids on *L*i*steria monocytogenes s*urvival: evidence for a viable but non- culturable state in response to low ph. Food Control. 2009;2009:1141–1144.

[22] Asakura H, Kawamoto K, Haishima Y, et al. Differential expression of the outer membrane protein w (ompw) stress response in enterohemorrhagic *Escherichia coli* o157: h7corresponds to the viable but non-culturable state. Res Microbiol. 2008;159(9– 10):709–717. 18824229 10.1016/j.resmic.2008.08.005

[23] Kana BD, Gordhan BG, Downing KJ, et al. The resuscitation-promoting factors of *Mycobacterium tuberculosis* are required for virulence and resuscita- tion from dormancy but are collectively dispensable for growth *in vitro*. Mol Microbiol. 2008;67(3):672–684. 18186793 10.1111/j.1365-2958.2007.06078.x PMC2229633

[24] Del Campo R, Russi P, Mara P, et al. *Xanthomonas axonopodis* pv. *citri* enters the vbnc state after copper treatment and retains its virulence. FEMS Microbiol Lett. 2009;298(2):143–148. 19624747 10.1111/j.1574-6968.2009.01709.x

[25] Ghezzi JI, Steck TR. Induction of the viable but non-culturable condition in *Xanthomonas campestris* pv. *campestris* in liquid microcosms and sterile soil. FEMS Microbiol Ecol. 1999;30(3):203–208. 10525176 10.1111/j.1574-6941.1999.tb00648.x

[26] Kusumoto A, Asakura H, Kawamoto K. General stress sigma factor rpos influences time required to enter the viable but non-culturable state in *Salmonella enterica*. Microbiol Immunol. 2012;56(4):228–237. 22256797 10.1111/j.1348-0421.2012.00428.x

[27] Lange R, Hengge-Aronis R. Growth phase-regulated expression of bolA and morphology of stationary-phase *Escherichia coli* cells are controlled by the novel sigma factor sigma S. J Bacteriol. 1991;173(14):4474–4481. 1648559 10.1128/jb.173.14.4474-4481.1991 PMC208111

[28] Tao K, Makino K, Yonei S, et al. Purification and characterization of the *Escherichia coli* oxyr protein, the positive regulator for a hydrogen peroxide-inducible regulon. J Biochem. 1991;109(2):262–266. 1864839

[29] Wei Q, Minh PN, Dötsch A, et al. Global regulation of gene expression by oxyr in an important human opportunistic pathogen. Nucleic Acids Res. 2012;40(10):4320–4333. 22275523 10.1093/nar/gks017 PMC3378865

[30] Muela A, Seco C, Camafeita E, et al. Changes in *Escherichia coli* outer membrane subproteome under environmental conditions inducing the viable but nonculturable state. FEMS Microbiol Ecol. 2008;64(1):28–36. 18318713 10.1111/j.1574-6941.2008.00453.x

[31] Day AP, Oliver JD. Changes in membrane fatty acid composition during entry of *Vibrio vulnificus* into the viable but nonculturable state. J Microbiol. 2009;42(2):69–73. 15357297

[32] Signoretto C, Lleò MM, Tafi MC, et al. Cell wall chemical composition of *Enterococcus faecalis* in the viable but nonculturable state. Appl Environ Microbiol. 2000;66(5):1953–1959. 10788366 10.1128/aem.66.5.1953-1959.2000 PMC101439

[33] Asakura H, Panutdaporn N, Kawamoto K, et al. Proteomic characterization of enterohemorrhagic *Escherichia coli* o157: h7in the oxidation-induced viable but non-culturable state. Microbiol Immunol. 2007;51(9):875–881. 17895604 10.1111/j.1348-0421.2007.tb03969.x

[34] González-Escalona N, Fey A, Höfle MG, et al. Quantitative reverse transcription polymerase chain reaction analysis of *Vibrio cholerae* cells entering the viable but non-culturable state and starvation in response to cold shock. Environ Microbiol. 2006;8(4):658–666. 16584477 10.1111/j.1462-2920.2005.00943.x

[35] Weichart D, Kjelleberg S. Stress resistance and recov- ery potential of culturable and viable but noncultur- able cells of *Vibrio vulnificus*. Microbiology. 1996;142(Pt 4):845–853. 8936311 10.1099/00221287-142-4-845

[36] Anuchin AM, Mulyukin AL, Suzina NE, et al. Dormant forms of *Mycobacterium smegmatis* with distinct morphology. Microbiology. 2009;155(Pt 4):1071–1079. 19332809 10.1099/mic.0.023028-0

[37] Wong HC, Wang P. Induction of viable but noncul- turable state in vibrio parahaemolyticus and its sus- ceptibility to environmental stresses. J Appl Microbiol. 2004;96(2):359–366. 14723697 10.1046/j.1365-2672.2004.02166.x

[38] Rowe MTDG, Loughney CF, Loughney CF, et al. Development of an image system for the study of viable but non-culturable forms of campylobacter jejuni and its use to determine their resistance to disinfectants. Food Microbiol. 1998;15(5):491–498.

[39] Lleò MM, Benedetti D, Tafi MC, et al. Inhibition of the resuscitation from the viable but non-culturable state in *Enterococcus faecalis*. Environ Microbiol. 2007;9(9):2313–2320. 17686027 10.1111/j.1462-2920.2007.01345.x

[40] Schottroff F, Fröhling A, Zunabovic-Pichler M, et al. Sublethal injury and viable but non-culturable (vbnc) state in microorganisms during preservation of food and biological materials by non-thermal processes. Front Microbiol. 2018;9:2773. 30515140 10.3389/fmicb.2018.02773 PMC6255932

[41] Lahtinen SJ, Ahokoski H, Reinikainen JP, et al. Degradation of 16S rRNA and attributes of viability of viable but nonculturable probiotic bacteria. Lett Appl Microbiol. 2008;46(6):693–698. 18444975 10.1111/j.1472-765X.2008.02374.x

[42] Kuehl B, Marten SM, Bischoff Y, et al. Maldi-tof mass spectrometry-multivariate data analysis as a tool for classification of reactivation and non-culturable states of bacteria. Anal Bioanal Chem. 2011;401(5):1593–1600. 21769553 10.1007/s00216-011-5227-5

[43] Yeware A, Gample S, Agrawal S, et al. Using diphe- nyleneiodonium to induce a viable but non-culturable phenotype in *Mycobacterium tuberculosis* and its metabolomics analysis. PLoS One. 2019;14(8):e0220628. 31369628 10.1371/journal.pone.0220628 PMC6675104

[44] Noack S, Wiechert W. Quantitative metabolomics: a phantom? Trends Biotechnol. 2014;32(5):238–244. 24708998 10.1016/j.tibtech.2014.03.006

[45] Baffone W, Casaroli A, Citterio B, et al. *Campylobacter jejuni* loss of culturability in aqueous microcosms and ability to resuscitate in a mouse model. Int J Food Microbiol. 2006;107(1):83–91. 16290304 10.1016/j.ijfoodmicro.2005.08.015

[46] Ayrapetyan M, Williams T, Oliver JD. Relationship between the viable but nonculturable state and anti- biotic persister cells. J Bacteriol. 2018;2018:200. 10.1128/JB.00249-18 PMC6153661 30082460

[47] Lleò MM, Pierobon S, Tafi MC, et al. mRNA detection by reverse transcription-PCR for monitoring viability over time in an *Enterococcus faecalis* viable but non- culturable population maintained in a laboratory microcosm. Appl Environ Microbiol. 2000;66(10):4564–4567. 11010918 10.1128/aem.66.10.4564-4567.2000 PMC92344

[48] Maalej S, Denis M, Dukan S. Temperature and growth-phase effects on *Aeromonas hydrophila* survi- val in natural seawater microcosms: role of protein synthesis and nucleic acid content on viable but tem- porarily nonculturable response. Microbiology. 2004;150(Pt 1):181–187. 14702411 10.1099/mic.0.26639-0

[49] Bovill RAMB. Resuscitation of ‘non-culturable’ cells from aged cultures of campylobacter jejuni. Microbiology. 1997;143(Pt 5):1575–1581. 9168608 10.1099/00221287-143-5-1575

[50] Amel BK, Amine B, Amina B. Survival of *Vibrio fluvialis* in seawater under starvation conditions. Microbiol Res. 2008;163(3):323–328. 16870413 10.1016/j.micres.2006.06.006

[51] Mukamolova GV, Kaprelyants AS, Kell DB, et al. Adoption of the transiently non-culturable state–a bacterial survival strategy? Adv Microb Physiol. 2003;47:65–129. 14560663 10.1016/s0065-2911(03)47002-1

[52] Panutdaporn N, Kawamoto K, Asakura H, et al. Resuscitation of the viable but non-culturable state of *Salmonella enterica* serovar *oranienburg* by recom- binant resuscitation-promoting factor derived from *Salmonella typhimurium* strain lt2. Int J Food Microbiol. 2006;106(3):241–247. 16213054 10.1016/j.ijfoodmicro.2005.06.022

[53] Reissbrodt R, Rienaecker I, Romanova JM, et al. Resuscitation of *Salmonella enterica* serovar *typhimur- ium* and enterohemorrhagic *Escherichia coli* from the viable but nonculturable state by heat-stable entero- bacterial autoinducer. Appl Environ Microbiol. 2002;68(10):4788–4794. 12324321 10.1128/AEM.68.10.4788-4794.2002 PMC126406

[54] Chaveerach P, Ter Huurne AA, Lipman LJ, et al. Survival and resuscitation of ten strains of *Campylobacter jejuni* and *Campylobacter coli* under acid conditions. Appl Environ Microbiol. 2003;69(1):711–714. 12514068 10.1128/AEM.69.1.711-714.2003 PMC152468

[55] Gupte AR, De Rezende CL, Joseph SW. Induction and resuscitation of viable but nonculturable salmonella enterica serovar typhimurium dt104. Appl Environ Microbiol. 2003;69(11):6669–6675. 14602627 10.1128/AEM.69.11.6669-6675.2003 PMC262293

[56] Helaine S, Kugelberg E. Bacterial persisters: formation, eradication, and experimental systems. Trends Microbiol. 2014;22(7):417–424. 24768561 10.1016/j.tim.2014.03.008

[57] Nowakowska J, Oliver JD. Resistance to environmen- tal stresses by vibrio vulnificus in the viable but non- culturable state. FEMS Microbiol Ecol. 2013;84(1):213–222. 23228034 10.1111/1574-6941.12052

[58] Bamford RA, Smith A, Metz J, et al. Investigating the physiology of viable but non-culturable bacteria by microfluidics and time-lapse microscopy. BMC Biol. 2017;15(1):21. 29262826 10.1186/s12915-017-0465-4 PMC5738893

[59] Kozarov EV, Dorn BR, Shelburne CE, et al. Human atherosclerotic plaque contains viable invasive *Actinobacillus actinomycetemcomitans* and *Porphyromonas gingivalis*. Arterioscler Thromb Vasc Biol. 2005;25(3):e17–18. 15662025 10.1161/01.ATV.0000155018.67835.1a

[60] Li L, Michel R, Cohen J, et al. Intracellular survival and vascular cell-to-cell transmission of *Porphyromonas gingivalis*. BMC Microbiol. 2008;8(1):26. 18254977 10.1186/1471-2180-8-26 PMC2259307

[61] Kortebi M, Milohanic E, Mitchell G, et al. *Listeria monocytogenes* switches from dissemination to persis- tence by adopting a vacuolar lifestyle in epithelial cells. PLoS Pathog. 2017;13(11):e1006734. 29190284 10.1371/journal.ppat.1006734 PMC5708623

[62] Boaretti M, LleO MM, Bonate B, et al. Involvement of rpoS in the survival of *EscMrichi4 coli* in the viable but non-culturable state. Environ Microbiol. 2003 Oct;5(10):986–996. 14510852 10.1046/j.1462-2920.2003.00497.x

[63] Kong I-S, Bates TC, Hulsmann A, et al. Role of catalase and oxyR in the viable but nonculturable state of *Vibrio vulnificus*. FEMS Microbiol Ecol. 2004 Nov 1;50(3):133–142. 19712354 10.1016/j.femsec.2004.06.004

[64] Wang H-W, Otung C-H, Ma T-Y, et al. Roles of alkyl hydroperoxide reductase subunit C (AhpC) in viable but nonculturable *Vibrio parahaemolyticus*. App Environ Microbial. 2013;79:3734–3743. 10.1128/AEM.00560-13 PMC3675929 23563952

[65] Kim J-C, Oh E, Kim J, et al. Regulation of oxidative stress resistance in *Campylobacter jejuni*, a microaerophilic foodborne pathogen. Front Microbiol. 2015 Jul 29;6:751. 26284041 10.3389/fmicb.2015.00751 PMC4518328

[66] Darcan C, Ozkanca R, Idil O, et al. Viable but non-culturable state (VBNC) of *Escherichia coli* related to EnvZ under the effect of pH, starvaton and 011110tic stress in sea water. Pol J Microbiol. 2009;58(4):307–317. 20380141

[67] Shleeva M, Mukamolova GV, Young M, et al. Formation of ‘non-culturable’ cells of *Mycobacterium smegmatis* in stationary phase in response to growth under suboptimal conditions and their Rpf-mediated resuscitation. Microbiology. 2004;150:1687–1697. 15184555 10.1099/mic.0.26893-0

[68] Ramamurthy T, Ghosh A, Pazhani GP, et al. Current perspectives on viable but non-culturable (VBNC) PATHOGENIC BACTERIA. Front Public Health. 2014 Jul 31;2:103. eCollection 2014. 25133139 10.3389/fpubh.2014.00103 PMC4116801

[69] Su X, Chen X, Hu J, et al. Exploring the potential environmental functions of viable but non-culturable bacteria. World J Microbiol Biotechnol. 2013 Dec;29(12):2213–2218. Epub 2013 Jun 4. 23733177 10.1007/s11274-013-1390-5

[70] Su X, Guo L, Ding L, et al. Induction of viable but nonculturable state in rhodococcus and transcriptome analysis using RNA-seq. PLoS One. 2016 Jan 25;11(1):e0147593. eCollection 2016. 26808070 10.1371/journal.pone.0147593 PMC4725852

[71] Mir M, Asong J, Li X, et al. The extracytoplasmic domain of the *Mycobacterium tuberculosis* Ser/Thr kinase PknB binds specific muropeptides and is required for PknB localization. PLoS Path. 2011;7:e1002182. 10.1371/journal.ppat.1002182 PMC3145798 21829358

[72] Li Y, Chen J, Zhao M, et al. Promoting resuscitation of viable but nonculturable cells of *Vibrio harveyi* by a resuscitation-promoting factor-like protein YeaZ. J Appl Microbiol. 2017 Feb;122(2):338–346. 27966258 10.1111/jam.13342

[73] Liu Y, Gilchrist A, Zhang J, et al. Detection of viable but nonculturable Escherichia coli O157: h7bacteria in drinking water and river water. Appl Environ Microbiol. 2008 Mar;74(5):1502–1507. Epub 2008 Jan 18. 18203853 10.1128/AEM.02125-07 PMC2258616

[74] Zhou W, Wang K, Hong W, et al. Development and application of a simple “Easy To Operate” propidium monoazide-crossing priming amplification on detection of viable and viable but non-culturable cells of O157 *Escherichia coli*. Front Microbiol. 2020 Sep 25;11:569105. eCollection 2020. 33101241 10.3389/fmicb.2020.569105 PMC7546352

